# Safety of oral nemonoxacin: A systematic review of clinical trials and postmarketing surveillance

**DOI:** 10.3389/fphar.2022.1067686

**Published:** 2022-12-09

**Authors:** Jinyi Yuan, Xiaoping Zhang, Jing Chen, Yueyuan Zhang, Fengjia Zhu, Haihui Huang

**Affiliations:** ^1^ Institute of Antibiotics, Huashan Hospital, Fudan University, Shanghai, China; ^2^ Researchand Development Center, Zhejiang Medicine Co., Ltd., Zhejiang, China

**Keywords:** nemonoxacin, non-fluorinated quinolone, safety, postmarketing surveillance, systematic review

## Abstract

**Background:** Postmarketing safety analysis is an effective supplement for new drugs in clinical practice. Therefore, we aimed to systematically assess the safety of oral nemonoxacin malate, the first approved C-8-methoxy non-fluorinated quinolone, in clinical studies and *via* postmarketing safety surveillance.

**Methods:** We electronically and manually searched and screened safety data (including premarketing and postmarketing data) of oral nemonoxacin from clinical registries. We standardized and summarized the reported adverse events according to the Medical Dictionary for Regulatory Activities System Organ Class and Preferred Terms. We summarized and reported the number and frequency (%) of the AEs and serious AEs in patients with community-acquired pneumonia and in specific patients.

**Results:** Three Phase II/III comparator studies (*n* = 670, nemonoxacin), one Phase IV study (*n* = 461), two special population pharmacokinetic studies (*n* = 40), four observational studies (*n* = 1,852), and one 5-year postmarketing surveillance project (*n* = 257,420) were included in this study. The Phase II/III studies showed that the commonly reported drug-related AEs were similar for oral 500 mg nemonoxacin and levofloxacin treatments, which mainly included increased alanine aminotransferase levels (4.4% vs. 2.5%), neutropenia (2.5% vs. 4.4%), nausea (2.5% vs. 1.6%), and leukopenia (2.3% vs. 3.2%). No drug-related deaths were reported. Postmarketing safety surveillance revealed that known adverse drug reaction characteristics were generally unchanged. Pharmacokinetic data suggested that dose adjustment was not necessary in elderly patients, which was confirmed by a Phase IV study in an elderly population, in patients with renal impairment with CLcr ≥50 ml/min, and in those with mild-to-moderate hepatic impairment.

**Conclusion:** Clinical trial data of approximately 1,450 patients and postmarketing data of >257,420 patients suggest that nemonoxacin is generally well tolerated and can be a suitable alternative to fluoroquinolones for patients with CAP.

## 1 Introduction

Nemonoxacin, a novel C-8-methoxy non-fluorinated quinolone, shows excellent microbiological efficacy and broad-spectrum antibacterial activity against most gram-positive cocci (including penicillin-resistant *Streptococcus pneumoniae* and methicillin-resistant *Staphylococcus aureus*), atypical pathogens, and most gram-negative bacteria (Chen et al., 2009; Lauderdale et al., 2010; Demei et al., 2015). The C-8 methoxy substituent of nemonoxacin targets topoisomerase IV and II, and substantially improves the activity spectrum and reduces the mutant selection (Arjona et al., 2009; Cheng et al., 2019). The absence of the C-6 fluorine moiety in the quinolone structure of nemonoxacin may be associated with a reduced incidence of toxic side effects (Barry et al., 2001). Nemonoxacin was approved by the National Medical Products Administration (NMPA) in 2016 and is indicated for community-acquired pneumonia (CAP) treatment (Poole, 2014). The recommended regimen for oral nemonoxacin is 500 mg once daily. In China, more than 765,000 patients have received nemonoxacin in the past 5 years (source: Pharmarket).

Evidence from preclinical studies shows that nemonoxacin is well tolerated, with the maximum dose being 1,500 mg single or 1000mg daily for 6 days. The most common treatment-emergent AEs are mild and include nausea, vomiting, leukopenia, and abnormal liver function (Lin et al., 2010). Even at a high dose of 750 mg, nemonoxacin has not shown a statistical difference in safety compared with levofloxacin. However, it is essential to monitor the safety profile of newly approved drugs in the postmarketing environment, which is the primary source of safety information. For example, fluoroquinolones are efficient and safe antibiotics (Mandell and Tillotson, 2002), with only mild and transient AEs. However, several rare but potentially severe AEs have been reported recently, which have led to license suspension, voluntary withdrawal, and restricted use of specific agents. These events highlight the importance of continued analysis of postmarketing surveillance data to identify rare but serious adverse drug reactions (ADRs) that cannot be observed in clinical trials because of the limited number of patients.

Postmarketing surveillance data includes information from clinical trials and investigator-initiated trials, but it is not limited to rigorous prospective data and also contains spontaneous reporting, social media, published literature, market research, and reports by regulatory authorities. Considering this information, we aimed to comprehensively evaluate the safety of oral nemonoxacin based on the data from clinical experiences, including Phase II/III clinical trials and postmarketing sources.

## 2 Materials and methods

### 2.1 Search strategy

The study was approved by Huashan Hospital Fudan University Ethics Committee (Approval number: (2017-366)). We searched all related studies, including information on the safety of oral nemonoxacin from the clinical trial register website, published papers, published conference reports, and unpublished clinical research reports froma sponsor database. We also searched postmarketing surveillance and a 5-year postmarketing surveillance management system for safety monitoring from the NMPA, including information from voluntary reporting, literature sources, and regulatory authorities, through 31 December 2021. Related data from published and unpublished evidence and pharmacokinetic studies were also screened and collected *via* a manual search.

**FIGURE 1:**
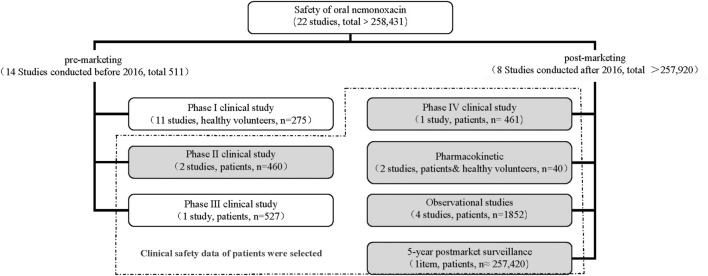
All safety results of oral nemonoxacin.

A total of 22 complete safety-related studies were searched. The study data in this paper were selected from 11 studies results on patients (Figure 1).

### 2.2 Participants

The participants in Phase II/III and Phase IV studies were CAP patients over 18 years old. Main inclusion criteria: 1) fever or white blood cell (WBC) count >9,500/mm^3^, or neutrophil count >70%, and at least two of the following: cough with purulent sputum, dyspnea or tachypnea, chest pain, and evidence of pulmonary consolidation; 2) have evidence of new pulmonary exudation or infiltrate (within 48 h before enrolment). Main exclusion criteria: 1) with severe community-acquired pneumonia; 2) simple viral pneumonia, aspiration pneumonia, or hospital acquired pneumonia; 3) <1,500 neutrophils/mm^3^ within 48 h before randomization; 4) treatment with systemic antimicrobial agent for more than 24 h and within 72 h before enrolment;5) current or anticipated long-term use (>2 weeks) of prednisone 20 mg per day or equivalent.

Two special pharmacokinetic (PK) studies enrolled 20 healthy volunteers normal liver and renal function, 10 patients with moderate liver damage (Child-Pugh Class B) and 10 patients with severe renal impairment or end-stage renal disease (eGFR≤30 ml/min/1.73 m^2^).

Observational studies and 5-year postmarketing surveillance focused on patients who had been treated with nemonoxacin and had no specific inclusion criteria.

### 2.3 Data selection

Reports and data on any AEs or safety issues of nemonoxacin, including research and surveillance reports and data, were identified and included. The researchers independently reviewed and evaluated the reports, any discrepancies were discussed, and if no agreement could be reached, a third researcher made the final decision.

### 2.4 Data extraction and safety evaluation

Information on the study type (Phase II, Phase III, Phase IV, PK, or observational study), study design, duration, dosage, comparison drugs, AEs, AE-related organ or system, degree of AEs (including drug-related, those that led to study drug discontinuation, and severe AE (SAE)), number and percentage of AEs, and the age and sex of the patients reporting AEs was extracted.

In clinical trials, safety was assessed by monitoring AE incidence and severity, vital sign assessments, cardiac assessments, laboratory evaluations, physical examinations, and concomitant medication usage. AE reporting and objective clinical data were evaluated whenever available from postmarketing and early access programs. All reported AEs were standardized and summarized according to the Medical Dictionary for Regulatory Activities System Organ Class and Preferred Terms. Numbers and percentages of subjects reporting AEs were calculated. The AE data were further classified according to characterized AEs of special interest, including gastrointestinal, hepatic, electrocardiograph (ECG), nervous, and skin or subcutaneous tissue disorders; laboratory test abnormalities; and other reported clinical concerns.

### 2.5 Statistical analysis

We summarized the number and frequency (%) of AEs and SAEs in patients using nemonoxacin. Furthermore, we summarized the number and frequency (%) of drug-related AEs, drug-related AEs leading to study drug discontinuation, SAEs related to the study drug, and deaths. The number and percentage of drug-related AEs with an incidence of ≥1% in different systems and organs and the sex of patients reporting AEs were summarized, and the number and percentage of elderly patients and patients with renal or hepatic impairment were calculated.

SPSS 26 statistical software was used for data analysis, rate difference and 95%CI were used to compare inter-group rates.

## 3 Results

We included three Phase II/III comparator studies with levofloxacin conducted in South Africa, Chinese mainland, and Taiwan, one Phase IV study, two postmarketing PK studies, four observational studies (the results of two studies have been published) (Zhao et al., 2020; Wenjin et al., 2021), and data from nearly 257,420 patients from an ADR monitoring report from Mainland China (Table 1). Additionally, no single case of adverse reactions was reported. The dosage was mainly 500 mg/d or 750 mg/d for 7–10 days (Cheng et al., 2019). Most of the patients experienced CAP, two PK studies included patients with moderate hepatic impairment, severe renal impairment, or healthy volunteers, and the sample size ranged from 121 to 356 for the Phase II/III studies and from 337 to 583 for Phase IV and observational studies.

**TABLE 1:** Overview of clinical studies and postmarketing surveillance.

Study phase and locations		Design, treatment dosage, and duration	No. of patients with nemofloxacin	No. of patients with LEVO	Participants
Phase II/III clinical study (Cheng et al., 2019)	Phase II; Chinese mainland (NCT01537250)	Randomized double-blind, multicenter; 500 or 750 mg/d, 7–10 days	500 mg: 62 + 12 (cases of PK study) 750 mg: 59 + 6 (cases of PK study)	56	Patients with CAP
	Phase II; Taiwan, South Africa (NCT00434291)	Randomized double-blind, multicenter; 500 or 750 mg/d, 7 days	500 mg: 89,750 mg: 86	90	Patients with CAP
	Phase III; Chinese mainland, Taiwan (NCT01529476)	Randomized double-blind, multicenter; 500 mg/d, 7–10 days	500 mg: 356	171	Patients with CAP
Postmarketing surveillance	Phase IV; Chinese mainland	Open-label, single-arm, multicenter; 500 mg/d, 7–10 days	461	—	Patients with CAP
	PK; Chinese mainland (Xiaoyong et al., 2020)	Pharmacokinetic, open-label, single-center; single-dose 500 mg	20	—	Patients with moderate hepatic impairment and healthy volunteers
	PK; Chinese mainland (Li et al., 2021)	Pharmacokinetic, open-label, single-center; single-dose 500 mg	20	—	Patients with severe renal impairment and healthy volunteers
	Observational studies; Chinese mainland, a total of 4 studies (Zhao et al., 2020; Wenjin et al., 2021)	500 mg/d, 7–10 days (main)	Total: 1852; study 1: 444; study 2: 337; study 3: 583; study 4: .488	—	Patients with CAP (main)
	ADR monitoring; Chinese mainland	500 mg/d, 7–10 days (main)	Total number ≈257,420	—	Patients with CAP (main)

Inter-group rate difference and 95%CI results showed that unless the incidence of AE and ADR related to Gastrointestinal disorders in the 750mg nemonoxacin group, there was no significant difference between nemonoxacin group and levofloxacin group (Table 2, 3).

**TABLE 2:** Safety summary of nemonoxacin in Phase II/III clinical studies.

AEs incidences	NEMO-500 mg (*n* = 519)	NEMO-750 mg (*n* = 151)	NEMO total (*n* = 670)	LEVO-500 mg (*n* = 317)	RD [95%CI]	
					NEMO-500 mg VS. LEVO-500 mg	NEMO-750 mg VS. LEVO-500 mg
AEs^a^	193 (37.2)	82 (54.3)	275 (41.0)	123 (38.8)	1.6 [−5.2; 8.4]	15.5 [5.9; 25.1]
Drug-related AEs	119 (22.9)	45 (29.8)	164 (24.5)	68 (21.5)	1.5 [−4.3; 7.3]	8.4 [−0.2; 16.9]
Drug-related AEs led to study drug discontinuation	2 (0.4)	2 (1.3)	4 (0.6)	1 (0.3)	0.1 [−0.8; 0.9]	1.0 [−0.9; 2.9]
SAEs^b^	12 (2.3)	6 (4.0)	18 (2.7)	3 (0.9)	1.4 [−0.3; 3.0]	3.0 [−0.3; 6.3]
Drug-related SAEs	1 (0.2)	0	1 (0.1)	0	—	—
Deaths	1 (0.2)	1 (0.7)	2 (0.3)	0	—	—
Study drug-related AEs leading to deaths	0	0	0	0	—	—

^a^ AE, refers to unexpected and adverse medical events, including clinically significant clinical laboratory abnormalities, that are related to any medical measures in the study, whether related to the study medication or not. (Same definition in full text).

^b^ SAE refers to events occurring during the clinical trial that require hospitalization, prolong hospital stay, disability, affect work ability, endanger life or death, and cause congenital malformations. (Same definition in full text)

**TABLE 3:** Drug-related adverse effects with an incidence of ≥1% in Phase II/III clinical studies.

Drug-related AEs	NEMO-500 mg (*n* = 519)	NEMO-750 mg (*n* = 151)	NEMO total (*n* = 670)	LEVO-500 mg (*n* = 317)	RD [95%CI]	
					NEMO-500 mg VS. LEVO-500 mg	NEMO-750 mg VS. LEVO-500 mg
Investigations^a^	**59 (11.4)**	**15 (9.9)**	**74 (11.0)**	**32 (10.1)**	**1.3 [-3.0; 5.6]**	**0.2 [-5.6; 6.0]**
Alanine aminotransferase increased	23 (4.4)	0	23 (3.4)	8 (2.5)		
Aspartate aminotransferase increased	10 (1.9)	1 (0.7)	11 (1.6)	3 (0.9)		
Gamma-glutamyltransferase increased	7 (1.3)	0	7 (1.0)	4 (1.3)		
Electrocardiogram - prolonged QT^b^	4 (0.8)	4 (2.6)	8 (1.2)	5 (1.5)		
Gastrointestinal disorders^a^	**31 (6.0)**	**16 (10.6)**	**47 (7.0)**	**14 (4.4)**	**1.6 [-1.5; 4.6]**	**6.2 [0.8; 11.6]**
Abdominal discomfort	5 (1.0)	2 (1.3)	7 (1.0)	1 (0.3)		
Upper abdominal pain	0	2 (1.3)	2 (0.3)	0		
Diarrhea	7 (1.3)	2 (1.3)	9 (1.3)	2 (0.6)		
Nausea	13 (2.5)	9 (6.0)	22 (3.3)	5 (1.6)		
Vomiting	6 (1.2)	4 (2.6)	10 (1.5)	7 (2.2)		
Nervous system disorders^a^	**14 (2.7)**	**4 (2.6)**	**18 (2.7)**	**6 (1.9)**	**0.8 [-1.2; 2.9]**	**0.8 [-2.2; 3.7]**
Dizziness	10 (1.9)	3 (2.0)	13 (1.9)	3 (0.9)		
Headache	5 (1.0)	2 (1.3)	7 (1.0)	3 (0.9)		
Blood and lymphatic system disorders^a^	**14 (2.7)**	**13 (8.6)**	**26 (3.9)**	**16 (5.0)**	**2.3 [-0.4; 5.1]**	**3.6 [-1.5; 8.6]**
Leukopenia^c^	12 (2.3)	7 (4.6)	19 (2.8)	10 (3.2)		
Neutropenia^d^	13 (2.5)	13 (8.6)	26 (3.9)	14 (4.4)		
Thrombocytosis^e^	4 (0.8)	4 (2.6)	8 (1.2)	2 (0.6)		
Skin and subcutaneous tissue disorders^a^	**6 (1.2)**	**1 (0.7)**	**7 (1.0)**	**5 (1.6)**	**0.4 [-1.2; 2.1]**	**0.9 [-1.0; 2.8]**
Rash	2 (0.4)	0	2 (0.3)	4 (1.3)		

^a^ List the total incidence.

^b^ Includes prolonged QT, and prolonged corrected QT, intervals.

c Includes decreased white blood cells count and leukopenia.

^d^ Includes decreased neutrophil percentage or count and neutropenia.

^e^ Includes increased platelet count and thrombocytosis.

The AE incidence in bold is calculated as SOC and is the sum of the incidence of AE for the corresponding PT.

### 3.1 Safety analysis of phase II/III studies

All three Phase II/III clinical studies were randomized clinical trials with a total of 987 patients randomized to receive nemonoxacin or levofloxacin; the treatment groups were balanced in terms of race, sex, age, weight, body mass index, baseline renal and liver functions, and other potential confounders. All randomized patients who received at least one dose of the study drug and only AEs reported after the treatment were included.

Overall, the incidence of AEs, SAEs, SAEs related to the study drug, discontinuation of study drug, and death due to adverse reactions were similar in the 500 mg nemonoxacin group (37.2%, 2.3%, 0.2%, 0.4%, and 0%, respectively) and the 500 mg levofloxacin group (38.8%, 0.9%, 0%, 0.3%, and 0%, respectively). There was a higher incidence of AEs in the 750 mg-nemonoxacin group (54.3%, 4.0%, 0%, 1.3%, and 0%, respectively) (Table 2). The most commonly reported AEs were comparable between the nemonoxacin and levofloxacin groups (Table 3). The drug-related AEs in the 500 mg nemonoxacin group and the 500 mg levofloxacin group were investigations (11.4% vs. 10.1%), gastrointestinal disorders (6.0% vs. 4.4%), nervous system disorders (2.7% vs. 1.9%), blood and lymphatic disorders (2.7% vs. 5.0%), and skin disorders (1.2% vs. 1.6%). Only two cases were severe, and >99.5% were mild and tolerable. The 750 mg-nemonoxacin group had higher incidences of AEs and SAEs.

### 3.2 Postmarketing safety surveillance

#### 3.2.1 Phase IV study

A total of 465 patients with CAP (49.2% men, average age 43.5 years) were enrolled in a Phase IV study. Of the 461 patients included in the safety analysis, 272 AEs related to the drug occurred in 117 patients. The incidence of ADR was 25.4% (Table 4), which was slightly higher than that in Phase II/III studies (22.9%). However, the severity of drug-related AEs and the types of AEs were similar to those of Phase II/III clinical trials, and of these, 92.3% were mild and transient (Table 5).

**TABLE 4:** Summary of nemonoxacin 500 mg in postmarketing surveillance.

Characteristics	Phase IV (China mainland) (*n* = 461)	PK study 1		PK study 2		PMOS1 (*n* = 444)	PMOS2 (*n* = 337)^a^	PMOS3 (*n* = 583)	PMOS4 (*n* = 488)	Database (*n*≈257, 420)^b^
		Hepatic impairment group (*n* = 10)	Healthy group (*n* = 10)	Renal impairment group (*n* = 10)	Healthy group (*n* = 10)					
Sex (%)										
Males	227 (49.2)	5 (50.0)	5 (50.0)	7 (70.0)	7 (70.0)	249 (56.1)	132 (40.9)	237 (40.7)	265 (54.3)	67 (57.3)
Females	234 (50.8)	5 (50.0)	5 (50.0)	3 (30.0)	3 (30.0)	195 (43.9)	191 (59.1)	346 (59.3)	223 (45.7)	50 (42.7)
Age (years)										
Mean (SD)	43.5 ± 15.9	54.10 ± 9.31	54.30 ± 11.25	45.38 ± 11.64	45.85 ± 10.95	53.59 ± 14.5	52 (34, 61)^c^	48.1 ± 16.0	53.3 ± 14.5	53.4 ± 17.4
Elderly (≥ 60 years)	95 (20.6)	4 (40.0)	3 (30.0)	1 (10.0)	1 (10.0)	158 (35.6)	--	≥65: 101 (17.3)	≥≥ 65: 125 (25.6)	≥70: 19 (16.2)
Treatment duration (day)										
Mean (SD)	8.5 ± 2.1	1	1	1	1	6.82 ± 2.4	8.24 ± 3.7	5.79 ± 2.1	7.6 ± 4.3	5.2 ± 3.8
AEs	200 (43.4)	6 (60.0)	3 (30.0)	8 (80.0)	2 (20.0)	6 (1.4)	6 (1.9)	11 (1.9)	2 (0.4)	117 (0.045)
Drug-related AEs	117 (25.4)	5 (50.0)	3 (30.0)	3 (30.0)	0	5 (1.1)	6 (1.9)	11 (1.9)	1 (0.2)	117 (0.045)
Drug-related AEs led to study drug discontinuation	13 (2.8)	0	0	0	0	2 (0.4)	0	0	1 (0.2)	77 (0.03)
SAEs	10 (2.2)	0	0	0	0	0	1 (0.3)	0	0	6 (0.002)
SAEs related to the study drug	2 (0.4)	0	0	0	0	0	1 (0.3)	0	0	6 (0.002)
Deaths	0	0	0	0	0	0	0	0	0	0

^a^ 14 patients were lost during the follow-up period;^9^.

^b^ Include a total of 257,420 patients with 2,630 feedbacks and 217 ICSRs, in 117 users.

^c^ Expressed as age median (P_25_, P_75_).

-- not reported.

**TABLE 5:** Drug-related adverse events with an incidence of ≥1% in Phase IV studies.

Drug-related AEs	Phase IV (China mainland) (*n* = 461)
Investigations	
Alanine aminotransferase increased	29 (6.3)
Aspartate aminotransferase increased	21 (4.6)
Gamma-glutamyltransferase increased	10 (2.2)
Electrocardiogram - Sinus bradycardia	6 (1.3)
Decreased white blood cells count	17 (3.7)
Neutropenia^a^	19 (4.1)
Thrombocytosis^b^	9 (2.0)
Nervous system disorders	
Dizziness	10 (2.2)
Gastrointestinal disorders	
Nausea	28 (6.1)
Diarrhea	7 (1.5)
Dry mouth	5 (1.1)
General disorders and administration site conditions	
Asthenia	6 (1.3)
Skin and subcutaneous tissue disorders	
Pruritus	5 (1.1)

#### 3.2.2 Observational studies

A total of 1852 patients from four studies (14 patients were lost during the follow-up period) treated with 500 mg qd from 2017 to 2020 were identified (Zhao et al., 2020; Wenjin et al., 2021). Twenty-three (1.25%) ADRs, including mild nausea, pruritus, and rash, were reported (Table 4). One patient experienced an unexpected adverse reaction, showing disorders of the nervous and musculoskeletal systems, which were accompanied by moderately numb lips and limbs and muscle spasms. No ADRs were observed in 46 patients with a longer treatment course than instructed; the longest was 30 days (Wenjin et al., 2021).

#### 3.2.3 5-Year postmarketing safety surveillance

As of 30 November 2020, there were 257,420 drug users in the 5-year postmarketing surveillance study. We identified 117 ADRs in the postmarketing surveillance, including five patient self-reports, 78 reports from medical institutes, and 34 reports from regulatory agencies. This data corresponds to an incidence rate of 0.45‰, mainly manifested as gastrointestinal disorders (35.0%), skin and subcutaneous tissue disorders (29.5%), and nervous system disorders (15.2%). Similar to the ADRs reported in clinical studies, the most common ones were nausea, pruritus, dizziness, rash, abdominal discomfort, and diarrhea.

The new ADRs reported in users after marketing involved 13 organs and systems. Six severe ADRs were reported, all of which were individual cases manifested as anaphylaxis, anaphylactic shock, insomnia/asthenia, fever, ocular hyperemia, and renal impairment (Table 6). Thus far, no disabling or potentially irreversible severe ADRs, including tendonitis and tendon rupture, peripheral neuropathy, and central nervous system effects, warned in the black box for systemic fluoroquinolone antibacterial drug instructions, have been observed (Chen et al., 2009).

**TABLE 6:** New reports of adverse drug reactions in postmarketing surveillance.

System/organ classification	New adverse reactions
Gastrointestinal disorders	Abnormal feces, gastrointestinal motility disorder, abnormal gastrointestinal sounds
Skin and subcutaneous tissue disorders	Swelling face (4), lip swelling (2), skin swelling (2), photosensitivity reaction, blister
Nervous system disorders	Insomnia (8), oral hypoesthesia (3), hypoesthesia, somnolence
General disorders and administration site conditions	Fever (2), chest discomfort (2), asthenia, chills, thirst, temperature intolerance, pain
Metabolism and nutrition disorders	Decreased appetite (7), hypoglycemia
Musculoskeletal and connective tissue disorders	Muscle weakness (2), joint pain
Immune system disorders	Hypersensitivity reaction (2), anaphylactic shock
Eye disorders	Ocular hyperemia, photophobia, lacrimation increased
Psychiatric disorders	Mental disorder, bruxism, agitation
Renal and urinary disorders	Polyuria
Cardiac disorders	Palpitation
Respiratory, thoracic and mediastinal disorders	Laryngeal edema
Investigations	Blood pressure decreased

^a^ Except the cases marked (whose occurrence times are indicated in brackets), the rest are single cases.

### 3.3 Special populations

#### 3.3.1 Patients with renal or hepatic impairment

Several Phase II/III and Phase IV studies enrolled a small number of patients with abnormal baseline or ongoing liver and kidney function issues. We summarized the adverse reactions in these patients and found that the incidence was no significant difference with that in the total cohort of enrolled patients (Table 7). The severity was similar; all were mild to moderate, except for one serious case unrelated to drug usage.

**TABLE 7:** Summary of nemonoxacin 500 mg safety in Phase II–IV studies stratified in patients with abnormal liver and kidney functions^a^.

AEs incidences	Phase II/III^b^					Phase IV (China mainland)				
	A (*n* = 28)	B (*n* = 23)	A + B (*n* = 17)	NEMO-500 mg (*n* = 519)	RD^c^ [95%CI]	A (*n* = 39)	B (*n* = 72)	A + B (*n* = 15)	NEMO-500 mg (*n* = 461)	RD^c^ [95%CI]
AEs	12 (42.9)	13 (56.5)	9 (52.9)	193 (37.2)	15.7 [−8.3; 39.8]	20 (51.3)	24 (33.3)	5 (33.3)	200 (43.4)	10.1 [−14.2; 34.3]
Drug-related AEs	10 (35.7)	9 (39.1)	7 (41.2)	119 (22.9)	18.3 [−5.4; 41.9]	13 (33.3)	16 (22.2)	3 (20.0)	117 (25.4)	5.4 [−15.3; 26.0]
Drug-related AEs led to study drug discontinuation	3 (10.7)	3 (13.0)	3 (17.6)	2 (0.4)	17.2 [−0.9; 35.4]	3 (7.7)	1 (1.4)	1 (6.7)	13 (2.8)	3.8 [−16.6; 8.9]
SAEs	1 (3.6)	1 (4.3)	1 (5.9)	12 (2.3)	3.6 [−7.7; 14.8]	1 (2.6)	0	0	10 (2.2)	—
Drug-related SAEs	0	0	0	1 (0.2)	—	1 (2.6)	0	0	2 (0.4)	—
Deaths	0	0	0	1 (0.2)	—	0	0	0	0	—
Study drug-related AEs leading to deaths	0	0	0	0	—	0	0	0	0	—

^a^ Abnormal liver and kidney function included: A with liver and/or kidney function abnormal baseline values that were clinically; B with ongoing liver and/or renal abnormalities; A + B means both A and B.

^b^ Based on Phase III, clinical and Phase II, clinical statistics numbered NCT01537250.

^c^ A + B VS. NEMO-500, mg.

Two PK studies on nemofloxacin showed a higher incidence of AEs in patients with mild and moderate hepatic or renal impairment with a creatinine clearance of ≥50 ml/min compared with the healthy population. Yet, they showed good tolerability and safety profiles of oral single-dose 500 mg nemonoxacin (Table 4) (Xiaoyong et al., 2020; Li et al., 2021). Study drug-related AEs manifested as Q-T interval prolongation, T wave change, white blood cell count decrease, total bilirubin increase, and skin pruritus, all of which were mild and transient, and the patients recovered without any treatment.

In addition, Two studies showed that there was no need to adjust the dose in patients with mild or moderate hepatic dysfunction and patients with creatinine clearance >50 ml/min. For patients with severe renal dysfunction (eGFR≤30 ml/min/1.73 m^2^) who are not on dialysis, 0.5 g every 36 h or 0.5 g every 48 h.

#### 3.3.2 Elderly patients

Analysis of age subgroups of Phase II/III studies and Phase IV clinical trials in Mainland China of 500 mg nemonoxacin treatment showed that the incidence of drug-related AEs was similar between age groups (<60 years: 27.9% vs. ≥60 years: 22.6%,95%CI: 1.7; 12.3) (Table 8). No serious ADRs were reported in the elderly group. One of the elderly patients who died of natural causes was reported to have SAE, but the investigator identified it as being unrelated to the usage of the drug.

**TABLE 8:** Summary of nemonoxacin 500 mg safety in Phase II–IV studies stratified by the age of patients.

AEs incidences	Phase II/III			Phase IV (China mainland)			Elderly total		
	Age <60 (*n* = 437)	Age ≥60 (*n* = 82)	RD [95%CI]	Age <60 (*n* = 366)	Age ≥60 (*n* = 95)	RD [95%CI]	Age <60 (*n* = 703)	Age ≥60 (*n* = 177)	RD [95%CI]
**AEs**	155 (35.4)	38 (46.3)	10.9 [−0.8; 22.6]	166 (45.4)	34 (35.8)	9.6 [−1.3; 20.5]	321 (45.7)	72 (40.7)	5.0 [−3.1; 13.1]
Drug-related AEs	99 (22.7)	20 (24.4)	1.7 [−8.4; 11.8]	97 (26.5)	20 (21.1)	5.5 [−3.9; 14.8]	196 (27.9)	40 (22.6)	5.3 [−1.7; 12.3]
Drug-related AEs led to study drug discontinuation	2 (0.5)	0	—	11 (3.0)	2 (2.1)	0.9 [−2.5; 4.5]	13 (1.8)	2 (1.1)	0.7 [−1.1; 2.6]
SAEs	10 (2.3)	2 (2.4)	0.2 [−3.5; 3.8]	6 (1.6)	4 (4.2)	2.6 [−1.7; 6.8]	16 (2.3)	6 (3.4)	1.1 [−1.8; 4.0]
Drug-related SAEs	1 (0.2)	0	—	1 (0.3)	1 (1.1)	0.8 [−1.3; 2.9]	2 (0.3)	1 (0.6)	0.3 [−0.9; 1.5]
Deaths	1 (0.2)	0	—	0	0	—	1 (0.1)	0	—
Study drug-related AEs leading to deaths	0	0	—	0	0	—	0	0	—

## 4 Discussion

This study screened and summarized safety data from Phase II/III clinical studies and postmarketing Phase IV studies and reports. Overall, nemonoxacin is generally well tolerated, with an overall ADR frequency similar to that of fluoroquinolone comparators. No potential novel or unexpected AEs have been reported for oral nemonoxacin. Additionally, nemonoxacin is tolerated in elderly patients and in those with renal impairment or mild to moderate hepatic impairment.

Since its launch in 2016, nemonoxacin has been administered to >765,000 patients (source: Pharmarket). Postmarketing surveillance has revealed no potential novel or unexpected low-frequency AEs, which supports the favorable safety profile of the drug in postmarking clinical use. In 2021, intravenous nemonoxacin was approved for marketing. In the future, ongoing clinical studies on the intravenous formulation will yield additional safety data, which might provide supplementary data regarding the safety of nemonoxacin. We integrated the premarketing data from Phase II and III reports in this study and observed that compared with the third-generation fluoroquinolone levofloxacin, nemonoxacin was more tolerable. Most AEs were mild to moderate and resolved without specific treatment. More importantly, postmarketing studies and surveillance reports in the broader population did not reveal any new warnings about serious ADRs, including the absence of several serious ADRs in the black box instructions for systemic fluoroquinolone antibacterial drug usage (Cheng et al., 2019). Therefore, based on current safety evidence, nemonoxacin is safe and lacks clinically significant safety concerns in terms of both premarketing and postmarketing data. However, owing to the limited data from 2016 to the present day, the safety of nemonoxacin needs to be monitored further. As the data accumulate, more information on nemonoxacin safety and efficacy will improve the user guidelines.

Moreover, we evaluated the safety of nemonoxacin in several specific populations. Similar ADRs and AEs were reported in elderly (aged >60 years) people and in patients with renal impairment or mild-to-moderate hepatic impairment. This evidence provides essential information regarding the use of nemonoxacin in these special populations. This data indicates that there is no need for dose adjustment in these special populations. Renal and liver functions are the main concerns of drug use; this finding provides guidelines for drug use in these special populations in clinical practice. However, we only included a single-dose PK study conducted in a small population; hence, this conclusion should be further evaluated and confirmed.

This study was limited by the inherent nature of reporting postmarketing data because reporting is spontaneous and includes sources such as published literature, market research, and public databases. These data may not be complete or capture details such as alternate etiologies, potential confounders, and time to the resolution of AEs, if applicable. The accuracy and validity of these data types are inferior to those from clinical trials. Thus, with the information obtained on individual cases, it is difficult to rule out the causal relationship between drug use and adverse reactions and reports because underlying diseases and complex medication combinations may exist in drug users. Moreover, the channel of information feedback could not be retraced. Therefore, postmarketing safety monitoring needs to be continued, and additional related information is necessary; our conclusions warrant further cumulative data from postmarketing surveillance.

## 5 Conclusion

This study comprehensively integrated current assessments of safety information and found that nemonoxacin, a novel non-fluorinated quinolone, is well tolerated and has an overall frequency of ADRs similar to that of fluoroquinolone comparators. Nemonoxacin is also well tolerated in elderly patients and in those with renal impairment or mild-to-moderate hepatic impairment, and no dose adjustment is necessary in these populations.

## Data Availability

The raw data supporting the conclusions of this article will be made available by the authors, without undue reservation.
